# Amalgam Phase-Down Part 1: UK-Based Posterior Restorative Material and Technique Use

**DOI:** 10.1177/2380084420978653

**Published:** 2020-12-10

**Authors:** O. Bailey, C.R. Vernazza, S. Stone, L. Ternent, A.-G. Roche, C. Lynch

**Affiliations:** 1 School of Dental Sciences, Newcastle University, Newcastle upon Tyne, UK; 2 Population Health Sciences Institute, Newcastle University, Newcastle upon Tyne, UK; 3 British Dental Association, London, UK; 4 University Dental School & Hospital, University College Cork, Cork, Ireland

**Keywords:** caries treatment, health services research, restorative dentistry, restorative materials, composite materials, clinical outcomes

## Abstract

**Introduction:**

A European Union amalgam phase-down has recently been implemented. Publicly funded health care predominates in the United Kingdom with the system favoring amalgam use. The current use of amalgam and its alternatives has not been fully investigated in the United Kingdom.

**Objectives:**

The study aimed to identify direct posterior restorative techniques, material use, and reported postoperative complication incidence experienced by primary care clinicians and differences between clinician groups.

**Methods:**

A cross-sectional survey was distributed to primary care clinicians through British dentist and therapist associations (11,092 invitations). The questionnaire sought information on current provision of direct posterior restorations and perceived issues with the different materials. Descriptive statistical and hypothesis testing was performed.

**Results:**

Dentists’ response rate was 14% and therapists’ estimated minimum response rate was 6% (total *N* = 1,513). The most commonly used restorative material was amalgam in molar teeth and composite in premolars. When placing a direct posterior mesio-occluso-distal restoration, clinicians booked on average 45% more time and charged 45% more when placing composite compared to amalgam (*P* < 0.0001). The reported incidences of food packing and sensitivity following the placement of direct restorations were much higher with composite than amalgam (*P* < 0.0001). Widely recommended techniques, such as sectional metal matrix use for posterior composites, were associated with reduced food packing (*P* < 0.0001) but increased time booked (*P* = 0.002).

**Conclusion:**

Amalgam use is currently high in the publicly funded sector of UK primary care. Composite is the most used alternative, but it takes longer to place and is more costly. Composite also has a higher reported incidence of postoperative complications than amalgam, but time-consuming techniques, such as sectional matrix use, can mitigate against food packing, but their use is low. Therefore, major changes in health service structure and funding and posterior composite education are required in the United Kingdom and other countries where amalgam use is prevalent, as the amalgam phase-down continues.

**Knowledge Transfer Statement:**

This study presents data on the current provision of amalgam for posterior tooth restoration and its directly placed alternatives by primary care clinicians in the United Kingdom, where publicly funded health care with copayment provision predominates. The information is important to manage and plan the UK phase-down and proposed phase-out of amalgam and will be of interest to other, primarily developing countries where amalgam provision predominates in understanding some of the challenges faced.

## Introduction

This is the first of 2 articles reporting a UK survey of primary care dentists and therapists investigating opinions, materials, and techniques used for direct posterior restoration provision. This article focuses on clinicians’ use of restorative techniques and materials, as well as experience of postoperative complications.

A global treaty prescribed the phase-down of amalgam on environmental grounds (Minamata Convention on Mercury 2013). The European Parliament agreed to the Regulation on Mercury (Regulation [EU] 2017/852 2017), which stipulated a phase-down beginning in July 2018. The regulation also specifies that the feasibility of a phase-out of amalgam, preferably by 2030, should be investigated.

Lynch and Wilson (2013) suggested the only viable directly placed alternative to amalgam, in the time frame specified for the amalgam phase-down, is composite resin (Lynch and Wilson 2013), although other options exist (Kielbassa et al. 2016), which include glass ionomer cements (GICs), or resin-modified GICs. There are a wide variety of composite materials with differing properties, and they can be placed with a variety of different techniques (Rosatto et al. 2015).

Evidence exists on posterior restorative material provision from around the world (Sunnegardh-Gronberg et al. 2009; Eklund 2010; Alexander et al. 2016), but health care provision generally differs from the primarily publicly funded health care with copayments for many (National Health Service [NHS]) provided in the United Kingdom.

Two surveys of material use for direct posterior restorations by general dentists (GDs) have recently been carried out in the United Kingdom. One survey looked at material provision for restoration of posterior teeth (Wilson et al. 2019), suggesting that composite has displaced amalgam as the most used dental restorative material in posterior permanent teeth in the United Kingdom. The sampling frame was limited, however, meaning the results may be less applicable across the United Kingdom. These results do not appear to correlate with the other survey results, which collected data from NHS GDs but was limited to Wales (Lynch et al. 2018). The Welsh survey does provide data on materials and techniques used but was not specific in assessing use of the different technique and material options currently available. Neither survey gives an indication of percentage use of the different available direct materials, with respondents being asked either which material was used most commonly (Wilson et al. 2019) or to rank their preferred choice of materials in specific situations (Lynch et al. 2018).

Amalgam was the most frequently used material to restore posterior teeth under NHS provision in Scotland in 2017–208 (Information Services Division 2017), and the expenditure on NHS amalgam fillings in England has been crudely estimated at £200 to £300 million from 2015 to 2016 (C.R. Vernazza and K. Carr, personal communication, 2018).

The most recent survey of NHS GDs in Wales showed a large majority felt that direct posterior composite provision was too expensive for NHS-funded dentistry and that there was a higher incidence of postoperative complications with posterior composite than amalgam restorations (Lynch et al. 2018), supporting the notion that composites are much more technique sensitive (Kielbassa et al. 2016), with differing techniques, materials, operators, and patient characteristics associated with differing outcomes (Demarco et al. 2012; Heintze and Rousson 2012; Schwendicke et al. 2018).

None of the current evidence relates to provision of restorations by dental therapists, who are a growing workforce in the United Kingdom (Centre for Workforce Intelligence 2014), or from dentists working in community dental services (CDS), who work with more challenging patients. Their patients commonly have behavioral difficulties and special requirements, which make achieving moisture control and higher levels of cooperation, as required for the placement of composite compared to amalgam restorations, very difficult, as evidenced by CDS dentists’ responses to the Scottish Dental Clinical Effectiveness Programme (SDCEP) consultation document on the phase-down of amalgam (M. West, personal communication, 2018).

Understanding current provision of direct posterior restorations in UK primary care is therefore critical to strategic planning for the potential phase-out of amalgam, and existing work does not provide sufficient detail. The objectives of this study were therefore (a) to identify and quantify current techniques, material use, and reported incidence of postoperative complications by UK dentists and therapists for placement of direct posterior restorations and (b) to determine any differences between subgroups.

## Methods

A cross-sectional e-survey was developed (accessible in the Appendix, with a link to the online questionnaire) consistent with STROBE (Strengthening the Reporting of Observational Studies in Epidemiology) guidelines, based on the recent Welsh survey (Lynch et al. 2018), alongside others identified in a literature review (Gilmour et al. 2009; Brunton et al. 2012; Alexander et al. 2016, 2017a, 2017b). It was modified, based on best practice questionnaire methodology (Dillman et al. 2014), to reduce survey error and to reflect the objectives of the study to obtain quantitative information on current techniques and materials used, rather than material preferences in particular situations. The study received a favorable ethical opinion from Newcastle University Research Ethics Committee (ref 7262/2018).

Open and closed questions were used, with utilization of clinical scenario vignettes and various Likert scales. The survey sought information on respondent demographics, education, current provision of direct posterior restorations (excluding localized cervical [class V]), and perceived issues with the different available materials. The questionnaire spanned a maximum of 24 screens containing 90 items, with 1 screen conditional on a previous response.

The questionnaire underwent initial usability testing through piloting, administered in paper form to internal and external academic dentist experts, as well as members of target respondent groups, using systematic form appraisals and “think-aloud” techniques (Geisen and Bergstrom 2017). It was then formatted electronically for use with the SmartSurvey online platform (www.smartsurvey.co.uk) before undergoing further piloting as previously described, alongside observational usability testing (Geisen and Bergstrom 2017), including ease of navigation with mobile devices. Modifications were made based on these processes to minimize survey error.

### Sample

A sample size calculation was performed, based on the core aspect of analysis, a multiple linear regression (MLR) investigating factors influencing time booked for placement of a mesio-occluso-distal (MOD) direct posterior composite with 21 independent variables. Various “rule-of-thumb” calculations exist for MLR minimum sample size, providing various estimates (Roscoe 1975), with 630 the largest obtained and therefore used (Pedhazur and Schmelkin 2013).

The questionnaire was then distributed by email to all British Dental Association (BDA) member GDs and CDS dentists, as well as all therapist members of the British Society of Dental Hygiene and Therapy (BSDHT) and the British Association of Dental Therapists (BADT) (11,092 invitations). A closed sampling frame was used for BDA members, which allowed tracking of respondents through the use of specific identifiers. This allowed the prevention of duplicate entries while allowing the use of targeted reminders and a monetary incentive of £100 for 1 respondent selected by random draw. Due to the systems in use, it was not possible to identify individual responders in the BSDHT and BADT groups; therefore, the sampling frame had to be open, and targeted reminders and incentivization were not possible. It was specified that the link should not be shared to limit the sampling frame to only those therapists receiving the invitational email. Two blanket reminders were sent to all 3 groups, with a link to the questionnaire attached. The questionnaire was launched February 14, 2019, and the deadline for response was March 31, 2019.

The first screen of the survey detailed its anonymity, the research purpose and team involved, data handling, and option and directions for opt-out. Consent was provided through a simple yes/no question after eligibility and understanding were similarly confirmed.

Survey data were received electronically and automatically captured by the BDA. Any identifiers were removed, and the anonymized data were passed securely to Newcastle University for analysis.

### Data Analysis

Data were cleaned, imported, and analyzed using Stata software (version 16; StataCorp LP). Subgroups (see Appendix data) were defined in relation to prior hypotheses. Data sets were assessed for normality of distribution graphically and using Shapiro-Wilk tests. Descriptive statistical testing was performed alongside 2-way hypothesis testing with χ^2^, Kruskal-Wallis, and Wilcoxon signed rank-sum tests, depending on the data.

Regression analyses were run with backward stepwise elimination. Best-fit models were selected by the lowest Bayesian information criterion value. Potential multicollinearity was assessed using variance inflation factors, with all obtained values less than 2.5. Multiple linear regressions were run to assess the impact of clinician and technique variables on private fee charged and appointment time booked for the placement of a direct posterior MOD composite. Logistic regressions were carried out to assess the impact of clinician and technique variables on reported low (0%–10%) incidences of postoperative food packing and sensitivity with direct posterior composite restorations. Data, samples, or models will be provided on request to the corresponding author.

## Results

In total, 1,570 responses were received. Fifty-four respondents were not suitable to participate in the study, answering negatively to one of the eligibility questions. This was mainly due to the respondents not currently practicing dentistry and placing direct restorations (*n* = 51). Three respondents were suitable but then failed to answer any further questions. A total of 1,513 usable responses were received. Dentists’ response rate was 14%, and therapists’ estimated minimum response rate was 6%. One respondent did not answer the final question, but all other remaining respondents did, giving a survey completion rate of 99.8% (of those who indicated their eligibility). A small minority of respondents gave contradictory answers (in the material usage section), which were excluded from analysis to reduce measurement error.

The minimum time taken for respondents to complete the questionnaire was 5 min (which was deemed sufficient time to complete the questionnaire), with a median value of 16 min.

Percentages are rounded to the nearest integer throughout. Direct posterior restorations throughout this article exclude localized cervical (class V) restorations.

### Demographics

The basic demographics are shown in Table 1.

**Table 1.** Summary Demographics.

Variable	Category		Frequency	Percent
Gender	Female		743	49
Clinician primary role	Dentist	NHS general (75%−100% NHS patient base)	617	41
		Mixed general (25%−74% NHS patient base)	194	13
		Private general (0%−24% NHS patient base)	509	34
		CDS	118	8
	Therapist		75	5
Primary dental qualification location	United Kingdom		1294	88
	EU (non−United Kingdom)		101	7
	Non-EU		81	5
Years qualified	≤2		57	4
	3−5		82	5
	6−10		159	11
	11−15		157	10
	16−20		195	13
	21−25		176	12
	26−30		195	13
	31−35		252	17
	≥36		239	16

CDS, community dental services; NHS, National Health Service.

Categorization of a dentist’s primary role was determined by the dominant number of sessions performed in general dentistry or CDS.

NHS and mixed GDs were evenly represented by gender, whereas private GDs had a greater proportion of males, and CDS dentists and therapists had a much greater proportion of females (Appendix Table 1), and the differences were statistically significantly different (χ^2^ *P* < 0.001).

Respondents whose primary dental qualification was European Union (EU; non−United Kingdom) or non-EU based mainly worked in general dentistry, with a lower proportion working in the CDS and none working as therapists (Appendix Table 2). The differences between groups were statistically significant (χ^2^ *P* = 0.001).

As dentists’ number of years of qualification increased, the proportion working as NHS GDs reduced and the proportion working as private GDs increased (Appendix Table 3), and this difference was statistically significant (χ^2^ *P* < 0.001).

### Material Use for Direct Posterior Restorations

Respondents were asked to state the percentage of premolars and molars that they restored with composite, amalgam, and other materials (Table 2). Composite was the most used directly placed material to restore premolar teeth, whereas amalgam was marginally the most used in molar teeth.

Only 6.7% of respondents used no amalgam and 0.4% of respondents used no composite for direct posterior restorations.

**Table 2.** Average Percentage Use of Amalgam, Composite, and Other Direct Materials (Glass Ionomer Cements/Resin-Modified Glass Ionomer Cements/Other) by Posterior Tooth.

	Average Use by Tooth (%)					
	Premolar			Molar		
Material	%	SD	Missing (%)	%	SD	Missing (%)
Composite	55	32	0.1	46	32	0.01
Amalgam	38	31	0.01	48	32	0.1
Other	6	10	0.1	6	9	0.3

Composite use in molar teeth increased as the clinicians’ number of years qualified increased from 32% (SD = 24) in those qualified for 0 to 5 y to 52% (SD = 33) in those qualified ≥26 y (Appendix Table 4). The differences were statistically significant (Kruskal-Wallis *P* = 0.0001).

The percentage of molar teeth restored directly with composite was lower in NHS GDs (26%; SD = 22) but higher in private GDs (73%; SD = 26) than therapists (41%; SD = 29), mixed GDs (45%; SD = 25), or CDS dentists (38%; SD = 28) (Appendix Table 5). The differences were statistically significant (Kruskal-Wallis *P* = 0.0001).

### Appointment Time and Fees Charged

Table 3 details the mean appointment time booked and mean private fees charged for different clinical scenarios. Wilcoxon signed rank tests showed that appointment time booked and private fee charged for a 3-surface MOD restoration in a molar tooth were statistically significantly higher (*P* < 0.0001) when comparing composite with amalgam as the restorative material. Similar statistical differences were shown for the 2-surface mesio-occlusal (MO) premolar restorations. Clinicians booked 45% more time and charged 45% more (as a private fee) to perform a direct MOD composite in a molar tooth than for the same restoration in amalgam. The ranges of appointment time booked and fees charged were wide.

**Table 3.** Appointment Time Booked and Private Fee Charged for Mesio-Occlusal (MO) Premolar and Mesio-Occluso-Distal (MOD) Molar Restorations.

Restoration	Material	Appointment Time Booked (min)				Cost (£)			
		Mean	SD	Range	Missing (%)	Mean	SD	Range	Missing (%)
Two-surface MO premolar	Composite	34	9	15−90	0.4	111.70	42	30−400	10
	Amalgam	24	7	10–60	4	77.60	34	13−350	18
Three-surface MOD molar	Composite	42	11	15−120	1	138.43	52	40–460	10
	Amalgam	29	8	5–60	4	95.50	43	18–450	18

NHS GDs booked shorter appointment times and private GDs longer appointment times than therapists, mixed GDs, and CDS dentists for direct MOD composite restorations. These differences were statistically significant (Kruskal-Wallis *P* = 0.0001) (Appendix Table 6).

### Direct Posterior Composite Technique and Material Use

Respondents were asked to indicate how often they used each composite technique, composite material, and bonding technique. They were given 8 options, including 0%, 100%, and 5 ranges in between. A not applicable option (N/A) was also included, which was only to be used if the clinician placed no composite restorations. These were analyzed and combined into the groupings shown in Appendix Tables 7, 8, and 9 under percentage use. The tables indicate the percentage of respondents who stated that they use the technique or material for each of the percentage use bands.

Rubber dam use for direct posterior composite restoration was generally low. Circumferential metal matrices were by far the most commonly used matrix. Use of a liner when placing a restoration in a tooth without a pulp exposure was variable, and wedges were commonly used when restoring a lost proximal surface (Appendix Table 7).

Incremental conventional composite placement was by far the most commonly used technique to directly restore a posterior tooth with composite compared with various bulk-fill options and nonincremental conventional placements (Appendix Table 8).

Use of a total-etch 2-step bonding technique was by far the most commonly used bonding strategy for posterior composite restoration placement (Appendix Table 9).

### Incidence of Postoperative Complications Encountered with Direct Posterior Restorations

Respondents were asked to indicate how often their patients experienced postoperative complications of sensitivity and food packing following placement of direct posterior composite and amalgam restorations. They were given 8 options, including 0%, 100%, and 5 ranges in between. A not applicable option (N/A) was also included, which was only to be used if the clinician placed no restorations of the indicated material. These were analyzed and combined into the groupings shown in Table 4 under the incidence (%) heading and its more specific variants. The percentage of respondents stating each frequency of complication groupings is shown.

**Table 4.** Clinician-Reported Incidence of Postoperative Problems Encountered following Direct Posterior Restoration Placement with Different Materials.

		Incidence (%)				
Postoperative Problem	Material	0–10	11–25	26–50	51–100	N/A
Sensitivity	Composite (*n* = 1,506)	52	29	12	5	1
	Amalgam (*n* = 1,507)	73	14	3	1	9
Food packing	Composite (*n* = 1,498)	58	29	9	4	1
	Amalgam (*n* = 1,508)	77	11	3	0	9

N/A, not applicable (i.e., the clinician does not use the material).

Wilcoxon signed rank tests showed statistically significantly higher clinician-reported incidences (*P* < 0.0001) of both food packing and sensitivity following direct posterior restoration with composite compared with amalgam. Forty-six percent reported sensitivity, and 42% reported food packing in more than 1 in 10 composite restorations placed, compared to 18% and 14%, respectively, with amalgam. Seventeen percent reported sensitivity, and 13% reported food packing in more than 1 in 4 composite restorations placed, compared to 4% and 3%, respectively, with amalgam (Table 4).

The N/A answers were removed and cross-tabulations performed, providing the following results.

Private GDs reported the lowest incidence of sensitivity following direct composite placement compared to other clinicians. Fifteen percent of therapists reported postoperative sensitivity in more than 1 in 2 direct composite restorations placed (Appendix Table 10). The differences were statistically significant (χ^2^ *P* < 0.001).

Private GDs reported the lowest incidence of food packing following direct composite placement compared to other clinicians (Appendix Table 11). The differences were statistically significant (χ^2^ *P* < 0.001).

Clinicians primarily using sectional metal matrices reported a much lower incidence of food packing following direct posterior composite restoration than those exclusively using circumferential matrices (Table 5). The difference was statistically significant (χ^2^ *P* < 0.001).

**Table 5.** Clinician-Reported Incidence of Food Packing and Sensitivity following Direct Posterior Composite Restoration with Various Matrix and Rubber Dam Use.

		Incidence after Composite Placement (%)			
Problem after Composite Placement	Technique Use	0–10	11–25	26–50	51–100
Food packing	100% circumferential metal matrix (*n* = 534)	50	32	12	6
	51%–100% sectional metal matrix (*n* = 266)	79	15	5	1
Sensitivity	0% rubber dam (*n* = 472)	49	32	13	6
	1%–10% rubber dam (*n* = 399)	55	28	13	4
	11%–75% rubber dam (*n* = 395)	52	32	12	5
	76%–100% rubber dam (*n* = 180)	64	22	9	5

Clinicians using rubber dam 76% to 100% of the time resulted in a lower incidence of reported sensitivity following direct posterior composite placement compared with other levels of use (Table 5). The difference was not statistically significant, however (χ^2^ *P* = 0.065).

As the clinicians’ number of years qualified increased, the incidence of postoperative food packing and sensitivity following amalgam and composite restorations reduced (Appendix Tables 12−15). The differences were all statistically significant (χ^2^ *P* < 0.001) except for food packing incidence after composite placement (χ^2^ *P* = 0.259).

### Bulk-Fill Composites

Sixty-eight percent of respondents reported having experience of using bulk-fill composites (*n* = 1,513). These clinicians had most experience of using flowable light-cured bulk-fill composites (53%). Smart Dentine Replacement (SDR; Dentsply) was by far the most commonly named material (42%). Interestingly, non-bulk-fill composites, compomers, GICs, and resin-modified GICs accounted for 8% of categorizable responses (Appendix Table 16).

Clinicians who had experience with using bulk-fill composites generally found them easier to place and were time saving but less aesthetic, with a majority neither agreeing nor disagreeing that they were more predictable or resulted in reduced postoperative sensitivity (Appendix Table 17).

### Regression Analyses

Full details of the regression analyses are shown in the Appendix information (Appendix Tables 18−21). In all cases, pseudo- or adjusted *R*^2^ values suggested a great deal of the variance was unexplained. However, significant factors in each model are discussed below.

A multiple linear regression analysis (*n* = 769; *P* < 0.001; adjusted *R*^2^ = 0.15) showed the factors statistically significantly associated with an increase in time booked for placing a direct posterior MOD composite (Appendix Table 18) were private GDs (6 min), therapists (5 min), and mixed GDs (4 min) compared to NHS GDs; high rubber dam users (6 min) compared to moderate users; primarily sectional metal matrix users (4 min); total-etch 3-step bond users (3 min); total-etch 2-step bond users (2 min) compared to self-etch 1-step bond users; and incremental composite users (2 min).

Factors statistically significantly associated with a decrease in time booked for placing a direct posterior MOD composite were clinicians who never use rubber dam (2 min) compared to moderate users and high-confidence MOD composite placers (2 min).

A multiple linear regression analysis (*n* = 711; *P* < 0.0001; adjusted *R*^2^ = 0.28) showed the factors statistically significantly associated with an increase in private fee charged for placing a direct posterior MOD composite (Appendix Table 19) were private GDs (£27.56) and mixed GDs (£12.91) compared to NHS GDs, high wedge users (£9.19), high-confidence MOD composite placers (£8.47), incremental composite users (£8.04), and appointment time booked for a direct posterior MOD composite (£1.43 per minute increase).

The factor statistically significantly associated with a decrease in private fee charged for placing a direct posterior MOD composite was clinicians who never use rubber dam (£10.53) compared to moderate use.

A logistic regression analysis (*n* = 770; *P* < 0.0001; pseudo-*R*^2^ = 0.11) showed the factors statistically significantly associated with a low incidence (0%−10%) of clinician-reported postoperative sensitivity following placement of a direct posterior composite (Appendix Table 20) were primarily composite users (combined premolar and molar composite usage >100%) (odds ratio [OR] = 2.3) and clinicians who never use a liner (OR = 1.8).

The factor statistically significantly associated with reduced likelihood of low incidence (11%−100%) of clinician-reported postoperative sensitivity following placement of a direct posterior composite was being a therapist compared to an NHS GD (OR = 0.4).

A logistic regression analysis (*n* = 768; *P* < 0.0001; pseudo-*R*^2^ = 0.09) showed the factors statistically significantly associated with a low incidence (0%−10%) of clinician-reported postoperative food packing following placement of a direct posterior composite (Appendix Table 21) were primarily composite users (OR = 2.8), primarily sectional metal matrix users (OR = 2.5), and incremental composite users (OR = 1.6).

## Discussion

This article details a UK-wide survey of dentists and therapists regarding their practice in placing direct posterior restorations. Composite is the most used material for direct restoration of premolars, whereas amalgam is in molar teeth. Amalgam use in posterior teeth in Australia, where private health care provision predominates, was 18% (Alexander et al. 2016). While this is different from the general data presented here, it does broadly correlate with data specific to private GDs. Composite use by private GDs is much higher than other primary care clinician groups, with the greatest disparity seen in relation to NHS GDs. Composite use in molar teeth increased as the clinicians’ number of years qualified increased, which shows a reverse correlation from data from other countries (Alexander et al. 2016) and directly refutes recent suggestions that the opposite was the case in the United Kingdom (Wilson et al. 2019). It is likely that this reflects the variation in composite provision in different types of practicing arrangements, with highest composite use seen by private GDs and the proportion of private GDs increasing with increasing age. Only 6.7% respondents used no amalgam at all, which is different from other countries, such as Australia (30%), where private health care provision predominates (Alexander et al. 2016).

Clinicians booked 45% more time and charged 45% more (as a private fee) to perform a direct MOD composite in a molar tooth than for the same restoration in amalgam. Dentists took 61% more time to place an occluso-proximal molar restoration in composite than amalgam from Welsh data (Lynch et al. 2018) and 43% from Irish data (Callanan et al. 2020a). Widely recommended posterior composite techniques, such as rubber dam use and sectional matrix use (Lynch et al. 2014), were low and have increased modestly in comparison to a UK survey of composite technique use from over 10 y ago (Gilmour et al. 2009). When used, these techniques were associated with an increased time taken to perform a composite restoration but a reduction in reported postoperative complications (not rubber dam). When placing posterior composite restorations, the best predictor of reported low postoperative food packing and sensitivity was if the clinician primarily used composite, while being a therapist was the best predictor of high reported postoperative sensitivity. Clinicians following current guidance in avoiding liner use under composite restorations (Blum and Wilson 2018) was associated with reduced reported postoperative sensitivity, further validating such an approach, although liner use was still common. On a positive note, the incidence of reported food packing associated with composite restorations has been hugely reduced in UK primary care over the past 10 y, whereas reported sensitivity is fairly similar (Gilmour et al. 2009).

However, clinician-reported postoperative incidence of sensitivity and food packing was much higher with composite than amalgam.

While bulk-fill composites are being adopted, there is still some confusion as to what constitutes a bulk-fill composite, which has implications for education.

Various potential sources of error and bias may have affected the results, with self-selection bias being the primary risk. In addition, there are concerns over recall bias, self-reporting, the possibility of repeat responses, a relatively low response rate, some small subgroup sizes, and potential differences in patients seen by different clinician groups in terms of disease prevalence, extent, and compliance, which may also have affected the results. Periodic repetition of the survey would be beneficial to support the findings, identifying trends and therefore health service and educational needs over time.

Clinical vignettes are limited in that they cover specific situations and do not take other “real-life” factors into account that potentially affect the generalizability of the data obtained.

The unique nature of publicly funded provision of dental services makes extrapolation of much of the data beyond the UK setting unsound, as evidenced by differences in material provision in other countries (Sunnegardh-Gronberg et al. 2009; Eklund 2010; Alexander et al. 2016; Callanan et al. 2020b), although primarily developing countries, which rely on amalgam to restore posterior teeth, may see similar postoperative issues for clinicians forced to use alternatives because of the phase-down. The data obtained from private dentists, however, may be generalized to many other countries where this mode of provision predominates and use of amalgam is permitted.

## Conclusion

Amalgam use in primary care is currently high, especially in the publicly funded sector, which is where the majority of direct posterior restoration provision lies. The alternatives are primarily composites, but there are a wide variety of materials and techniques being used under this banner. There is a much higher reported incidence of postoperative complications with composites, although time-consuming techniques, such as sectional matrix use, are associated with reduced reported postoperative food packing, although their use is currently low in the United Kingdom. High posterior composite usage is the best predictor of reduced reported postoperative complications, but posterior composites cost more and take longer to perform. This suggests that major changes in health service structure and funding and education on posterior composite technique are required in the United Kingdom and other countries where amalgam use is still prevalent, as the amalgam phase-down continues.

## Author Contributions

O. Bailey, contributed to conception, design, and data analysis, drafted the manuscript; C.R. Vernazza, contributed to conception, design, data analysis, and interpretation, drafted and critically revised the manuscript; S. Stone, L. Ternent, A.-G. Roche, contributed to conception, design, data analysis, and interpretation, critically revised the manuscript; C. Lynch, contributed to design, critically revised the manuscript. All authors gave final approval and agree to be accountable for all aspects of the work.

## Supplemental Material

sj-pdf-1-jct-10.1177_2380084420978653 – Supplemental material for Amalgam Phase-Down Part 1: UK-Based Posterior Restorative Material and Technique Use Click here for additional data file. Supplemental material, sj-pdf-1-jct-10.1177_2380084420978653 for Amalgam Phase-Down Part 1: UK-Based Posterior Restorative Material and Technique Use by O. Bailey, C.R. Vernazza, S. Stone, L. Ternent, A.-G. Roche and C. Lynch in JDR Clinical & Translational Research
