# Supplementary material for: Amalgam Phase-Down Part 1: UK-Based Posterior Restorative Material and Technique Use
Source: JDR Clin Trans Res. 2020 Dec 10;7(1):41–9. doi: 10.1177/2380084420978653 (PMC8674792; doi:10.1177/2380084420978653)
Supplement: sj-pdf-1-jct-10.1177_2380084420978653 – Supplemental material for Amalgam Phase-Down Part 1: UK-Based Posterior Restorative Material and Technique Use [file sj-pdf-1-jct-10.1177_2380084420978653.pdf]

Amalgam phase-down part 1: UK based posterior restorative material and technique use

Bailey OS, Vernazza CR, Stone S, Ternent L, Roche A-G, Lynch CD

Appendix

Questionnaire pg 1
Appendix results tables pg 19

Questionnaire

Online questionnaire available at:

https://www.smartsurvey.co.uk/s/preview/82YU5/A693DA6425DF4DAB4A113A34AEF0E2

A Newcastle University, BDA and BSDHT study on alternatives to dental amalgam

The phase-down of amalgam is currently a much-debated topic in dentistry in the UK. We would hugely value your input on this topic.

This questionnaire is a collaboration between Newcastle University, the British Dental Association (BDA) and the British Society of Dental Hygiene and Therapy (BSDHT). The data will be used in a PhD project that is being undertaken at Newcastle University. This will investigate the cost effectiveness of directly placed restorative materials, compared to dental amalgam. The results will be used by the BDA to campaign on this issue and to advise governments on the issues for dentists in relation to amalgam phase-down. The BSDHT will use them to inform policy.

This survey will assess current material use, and techniques employed in the direct (non-laboratory) restoration of posterior teeth. It will also assess the opinions of dentists and therapists surrounding this topic, so your participation would be greatly appreciated and is important for the validity of this study. We would like you to be as honest as possible about your individual practice and opinions.

The questionnaire should take around 10 minutes to complete. Only complete this questionnaire if you place direct posterior restorations and please do not forward the web link on to avoid sampling

errors.

Identifiable information will be separated from responses prior to transfer and analysis at Newcastle University, therefore all information will be anonymous. This study has ethical approval from Newcastle University.

The results of the survey and the cost effectiveness analysis will be submitted for publication in due course.

If you wish to opt-out of this survey at any point, please email Research@bda.org with "AM OPT-OUT" in the subject line.

GDPR statement

How the information will be used

The information is collected by the British Dental Association (BDA) to support the policy activity it undertakes on behalf of the profession, to provide evidence in a PhD project undertaken at Newcastle University and to inform BSDHT policy. All data will be used for research purposes only and any information you provide will be treated confidentially.

What happens to the data collected?

Data from all participants will be coded, combined and analysed independently. Parts of the study may also be submitted for publication. Direct quotes from the survey may be used in reports and publications but quotes will be anonymised to ensure that participants cannot be identified.

Storage of your personal data

All information you provide to us is stored on secure servers. The data that we collect from you will not be transferred to, or stored at, a destination outside the European Economic Area ("EEA"). Your personal data collected through this survey will be stored for up to seven years. Data will be stored on our servers and our survey platform which is SmartSurvey.

Access to information

You have the right to request a copy of the information we hold about you.

What do I need to do?

You are not required to take part in this study but your participation will help us to improve the working lives of dentists and therapists. Your information will be aggregated with the other respondents' information.

The data controller

For the purpose of the General Data Protection Regulation 2018 (the Act), the data controller is The British Dental Association of 64 Wimpole Street, London W1G 8YS.

The data processor

For the purpose of the Act, the data processors are both The British Dental Association and SmartSurvey Ltd of Unit 23, Basepoint Business Center, Tewkesbury, GL20 8SD. For more information, consult their Privacy Policy and Notice at https://www.smartsurvey.co.uk/privacy-policy, Part 2 covers Privacy of Survey Respondents.

If you are not happy

If you feel that we have mistreated the handling of your data please contact us in the first instance. If you are not satisfied with our response you are entitled to lodge a complaint with the Information Commissioner, Wycliffe House, Water Lane, Wilmslow SK9 5AF.

Thank you for taking the time to read this information

2. HOW TO NAVIGATE ...

To navigate the questionnaire, please use the Previous Page and Next Page buttons located at the bottom of each page.

Please do not use the back arrow of your web browser as this will exit the study.

In the eventuality that this happens, please go back to your email invitation and click once more on your SmartSurvey link.

I confirm that I have read and understand the purpose of this research and have had the opportunity to consider the information and my involvement. *

- ☐ Yes
- ☐ No

I understand that my involvement is voluntary and I consent to participating in this study. *

- ☐ Yes

☐ No

I currently practice dentistry and I place direct posterior restorations. *

☐ Yes

☐ No

4. FREQUENCY OF PLACEMENT OF DIRECT POSTERIOR RESTORATIONS

When definitively restoring premolar teeth (NOT class V or localised cervical) with directly placed materials, what percentage would you estimate you restore with? Sum total should equal 100%

Composite	
Amalgam	
GIC/RMGIC/Other	

When definitively restoring molar teeth (NOT class V or localised cervical) with directly placed materials, what percentage would you estimate you restore with? Sum total should equal 100%

Composite	
Amalgam	
GIC/RMGIC/Other	

5. TECHNIQUE

How often do you use the following techniques when placing direct posterior restorations (NOT class V or localised cervical) of the indicated materials? Only select 'not applicable' if you do not place any direct posterior restorations using the indicated material. Rubber dam

	0%	1-10%	11-25%	26-50%	51-75%	76-99%	100%	not applicable
Amalgam	☐	☐	☐	☐	☐	☐	☐	☐
Composite	☐	☐	☐	☐	☐	☐	☐	☐

Liner (in cavities with no obvious pulp exposure)

	0%	1-10%	11-25%	26-50%	51-75%	76-99%	100%	not applicable
Amalgam	☐	☐	☐	☐	☐	☐	☐	☐

Please specify materials used under amalgam

	0%	1-10%	11-25%	26-50%	51-75%	76-99%	100%	not applicable
Composite	☐	☐	☐	☐	☐	☐	☐	☐

Please specify materials used under composite

6. TECHNIQUE

How often do you use the following techniques when placing direct posterior restorations (NOT class V or localised cervical) of the indicated materials? Only select 'not applicable' if you do not place any direct posterior restorations using the indicated material. Matrix bands (when restoring a lost proximal surface) Circumferential metal (e.g. Siqveland, Toffelmire, Disposable types)

	0%	1-10%	11-25%	26-50%	51-75%	76-99%	100%	not applicable
Amalgam	☐	☐	☐	☐	☐	☐	☐	☐
Composite	☐	☐	☐	☐	☐	☐	☐	☐

Circumferential clear (e.g. Disposable types)

	0%	1-10%	11-25%	26-50%	51-75%	76-99%	100%	not applicable
Amalgam	☐	☐	☐	☐	☐	☐	☐	☐
Composite	☐	☐	☐	☐	☐	☐	☐	☐

Sectional metal (e.g. Palodent, Garrison)

	0%	1-10%	11-25%	26-50%	51-75%	76-99%	100%	not applicable
Amalgam	☐	☐	☐	☐	☐	☐	☐	☐
Composite	☐	☐	☐	☐	☐	☐	☐	☐

Sectional clear (e.g. Bioclear)

	0%	1-10%	11-25%	26-50%	51-75%	76-99%	100%	not applicable
Amalgam	☐	☐	☐	☐	☐	☐	☐	☐

	0%	1-10%	11-25%	26-50%	51-75%	76-99%	100%	not applicable
Composite	☐	☐	☐	☐	☐	☐	☐	☐

7. TECHNIQUE

How often do you use the following techniques when placing direct posterior restorations (NOT class V or localised cervical) of the indicated materials? Only select 'not applicable' if you do not place any direct posterior restorations using the indicated material. Wedge/s (when restoring a lost posterior proximal surface)

	0%	1-10%	11-25%	26-50%	51-75%	76-99%	100%	not applicable
Amalgam	☐	☐	☐	☐	☐	☐	☐	☐
Composite	☐	☐	☐	☐	☐	☐	☐	☐

8. TECHNIQUE

How often do you use the following materials when placing direct posterior composite restorations (NOT class V or localised cervical)? Only select 'not applicable' if you do not place any direct posterior restorations using the indicated material. Composite specific Bonding agents

	0%	1-10%	11-25%	26-50%	51-75%	76-99%	100%	not applicable
Separate etch (and rinse) + bond (in 1 bottle, 2-step) eg. Optibond Solo Plus	☐	☐	☐	☐	☐	☐	☐	☐
Separate etch (and rinse) + prime + bond	☐	☐	☐	☐	☐	☐	☐	☐

	0%	1-10%	11-25%	26-50%	51-75%	76-99%	100%	not applicable
(in 2 bottles, 3-step) eg. Optibond FL								
Self-etch (1 bottle) eg. Prompt-L-Pop, iBond	☐	☐	☐	☐	☐	☐	☐	☐
Self-etching primer + bond (2 bottles) eg. Clearfil SE II bond	☐	☐	☐	☐	☐	☐	☐	☐
Selective enamel etch technique (phosphoric acid on enamel only) with self-etching systems	☐	☐	☐	☐	☐	☐	☐	☐

9. TECHNIQUE

How often do you use the following materials when placing direct posterior composite restorations (NOT class V or localised cervical)? Only select 'not applicable' if you do not place any direct posterior restorations using the indicated material. Composite specific Composite material/s

	0%	1-10%	11-25%	26-50%	51-75%	76-99%	100%	not applicable
Flowable bulk-fill composite alone	☐	☐	☐	☐	☐	☐	☐	☐
Paste-like bulk-fill composite alone	☐	☐	☐	☐	☐	☐	☐	☐
Flowable bulk-fill composite capped with a conventional composite	☐	☐	☐	☐	☐	☐	☐	☐
Flowable bulk-fill composite capped with a paste-like bulk-fill composite	☐	☐	☐	☐	☐	☐	☐	☐

	0%	1-10%	11-25%	26-50%	51-75%	76-99%	100%	not applicable
Incrementally placed conventional (paste-like) composite	☐	☐	☐	☐	☐	☐	☐	☐
Non-incrementally placed conventional (paste-like) composite	☐	☐	☐	☐	☐	☐	☐	☐

10. TECHNIQUE

Do you have experience in using bulk-fill composites?

☐ Yes

☐ No

Name of bulk-fill composite/s used

I have found them ...

	Strongly agree	Agree	Neither agree nor disagree	Disagree	Strongly disagree
... easier to place than conventional composites	☐	☐	☐	☐	☐
... time saving compared to conventional composites	☐	☐	☐	☐	☐

	Strongly agree	Agree	Neither agree nor disagree	Disagree	Strongly disagree
... to have reduced post-operative sensitivity compared to conventional composites	☐	☐	☐	☐	☐
... to have more predictable outcomes than conventional composites	☐	☐	☐	☐	☐
... more aesthetic than conventional composites	☐	☐	☐	☐	☐

12. CLINICAL SCENARIOS

If you had to restore a moderately deep 2-surface mesio-occlusal cavity in an upper premolar with amalgam, how long an appointment would you book? In minutes

If you had to restore the same cavity with composite, how long an appointment would you book? In minutes

If you restored the tooth with composite privately, what fee would you charge? In £

If you restored the tooth with amalgam privately, what fee would you charge? In £

13. CLINICAL SCENARIOS

If you had to restore a deep 3-surface mesio-occlusal-distal cavity in a lower first molar with amalgam, how long an appointment would you book? In minutes

If you had to restore the same cavity with composite, how long an appointment would you book? In minutes

If you restored the tooth with composite privately, what fee would you charge? In £

If you restored the tooth with amalgam privately, what fee would you charge? In £

14. FEES

What would the percentage change in profitability be, in providing a posterior composite, rather than a posterior amalgam restoration under NHS provision? Only complete one of these two boxes please.

	Percentage change
Increase	
Decrease	

15. POST-TREATMENT PROBLEMS

How often do you see the following complications within one year when using the following materials to directly restore posterior teeth (NOT class V or localised cervical)? Only select 'not applicable' if you do not place any direct posterior restorations using the indicated material. Sensitivity

	0%	1-10%	11-25%	26-50%	51-75%	76-99%	100%	not applicable
Amalgam	☐	☐	☐	☐	☐	☐	☐	☐
Composite	☐	☐	☐	☐	☐	☐	☐	☐

Food packing (when restoring a proximal contact)

	0%	1-10%	11-25%	26-50%	51-75%	76-99%	100%	not applicable
Amalgam	☐	☐	☐	☐	☐	☐	☐	☐
Composite	☐	☐	☐	☐	☐	☐	☐	☐

16. KNOWLEDGE BASE

The Mercury Regulation that is now in force has as one of its aims a phase-down of the use of dental amalgam. Considerations for a potential 'phase-out' of the material are currently being considered at EU level.

We wish to understand the dentists’ and therapists’ knowledge and opinions of the 'phase-out' with the following three questions.

In which year is it intended that the possible 'phase-out' of amalgam ought to be complete by?

- ☐ 2020
- ☐ 2025
- ☐ 2030
- ☐ 2035
- ☐ 2040
- ☐ 2045
- ☐ 2050

In which patient groups should the use of amalgam be avoided according to current rules?

Over which period of time do you believe dental amalgam should be ‘phased-out’ in UK dental practice?

- ☐ Less than 5 years
- ☐ 5 – 9 years
- ☐ 10 – 19 years
- ☐ 20 – 29 years
- ☐ More than 30 years

17. YOUR OPINIONS

Please indicate to which level you agree or disagree with the following statements:

The 'phasing-out' of amalgam ...

	Strongly agree	Agree	Neither agree nor disagree	Disagree	Strongly disagree
... will impact on my ability to do my job	☐	☐	☐	☐	☐
... will lead to the need for more indirect restorations	☐	☐	☐	☐	☐
... will lead to more teeth being deemed unrestorable	☐	☐	☐	☐	☐
There is a lack of consensus on best practice when selecting direct alternative materials	☐	☐	☐	☐	☐
There is a lack of consensus on best practice in terms of technique when directly placing alternative materials	☐	☐	☐	☐	☐
My patients won't care	☐	☐	☐	☐	☐

18. YOUR OPINIONS

Please indicate to which level you agree or disagree with the following statements:

Alternative direct materials

	Strongly agree	Agree	Neither agree nor disagree	Disagree	Strongly disagree
Suitable directly placed alternatives to amalgam are available	☐	☐	☐	☐	☐
I feel up to date with current techniques and practices relating to placement of posterior composites	☐	☐	☐	☐	☐
Having to routinely place posterior composites would cause appointment delays in my practice	☐	☐	☐	☐	☐
Posterior amalgams last longer than directly placed posterior composites	☐	☐	☐	☐	☐
It takes me longer to remove a failed posterior composite restoration than a failed amalgam restoration of equivalent size	☐	☐	☐	☐	☐

19. YOUR OPINIONS

Please indicate to which level you agree or disagree with the following statements:

Alternative direct materials

Please indicate your confidence level ...

	No confidence	Low confidence	Moderate confidence	High confidence	Complete confidence
... in providing 2 surface direct posterior	☐	☐	☐	☐	☐

	No confidence	Low confidence	Moderate confidence	High confidence	Complete confidence
composite restorations involving a proximal surface					
... in providing 3 surface direct posterior composite restorations involving both proximal surfaces	☐	☐	☐	☐	☐
... in providing definitive 2 surface posterior GICs involving a proximal surface	☐	☐	☐	☐	☐
... in providing definitive 3 surface posterior GICs involving both proximal surfaces	☐	☐	☐	☐	☐
... when placing direct posterior composites with sub-gingival margins	☐	☐	☐	☐	☐
... when placing posterior amalgams with sub-gingival margins	☐	☐	☐	☐	☐
... when placing direct posterior composites in patients with limited cooperation	☐	☐	☐	☐	☐
... when placing posterior amalgams in patients with limited cooperation	☐	☐	☐	☐	☐

20. DEMOGRAPHICS

At which institution did you obtain your primary dental qualification?

In which year did you obtain your primary dental qualification?

Please indicate your professional role

- [ ] Dentist
- [ ] Therapist

21. DEMOGRAPHICS

Please indicate your gender

- [ ] Male
- [ ] Female
- [ ] Prefer not to say

Please indicate the number of sessions per week worked in (considering a morning a session, an afternoon a session and an evening a session)

	Number of sessions per week worked in the following settings
Hospital	
Community	

Number of sessions per week worked in the following settings

Specialist practice [ ]

General practice [ ]

Approximately, what proportion of your patients do you personally provide NHS care for?

- [ ] 100% (exclusively NHS patients)
- [ ] 75-99% NHS
- [ ] 50-74% NHS
- [ ] 25-49% NHS
- [ ] 1-24% NHS
- [ ] 0% (exclusively private patients)

22. TRAINING

Please select the appropriate box

	Yes	No	Unsure
Did you receive didactic instruction (e.g. lectures, seminars) in posterior composite placement as part of your dental school training?	☐	☐	☐
Did you receive clinical training in posterior composite placement as part of your dental school training?	☐	☐	☐
Since graduation have you attended CPD courses relating to the placement of posterior composites?	☐	☐	☐

Any further comments?

Appendix results tables

Appendix Table 1: Workforce by gender; female, male, or prefer not to say (PNTS)

**Clinician**		**Female (%)**	**Male (%)**	**PNTS (%)**
Dentist	NHS GD (n=615)	49	48	3
	Mixed GD (n=193)	49	48	3
	Private GD (n=505)	36	62	2
	CDS (n=118)	78	19	3
Therapist (n=75)		89	7	4

Appendix Table 2: Workforce by primary dental qualification location

**Clinician**		**Primary dental qualification location (%)**		
		UK	EU (non-UK)	Non-EU
Dentist	NHS GD (n=591)	84	9	7
	Mixed GD (n=190)	84	8	7
	Private GD (n=503)	89	6	5
	CDS (n=116)	95	3	3
Therapist (n=75)		100	0	0

Appendix Table 3: Workforce by years qualified

**Years Qualified**	**Clinician (%)**				
	Dentist				Therapist
	NHS GD	Mixed GD	Private GD	CDS	
0-5 (n=139)	63	10	7	6	13
6-15 (n=316)	53	12	15	7	13
16-25 (n=371)	42	15	32	9	2
≥26 (n=686)	30	13	49	8	1

Appendix Table 4: Percentage use of direct composite in molar teeth by years qualified

**Years qualified**	**Mean percentage of molar teeth restored with composite**	
	%	SD
0-5 (n=139)	32	24
6-15 (n=316)	40	28
16-25 (n=371)	46	33
≥26 (n=686)	52	33

Appendix Table 5: Percentage use of direct composite in molar teeth by clinician

**Clinician**	**Mean proportion of molar teeth restored with composite**	
	%	SD
NHS GD (n=617)	26	22
Mixed GD (n=193)	45	25
Private GD (n=509)	73	26
CDS dentist (n=118)	38	28
Therapist (n=75)	41	29

Appendix Table 6: Appointment time booked to place direct posterior mesio-occluso-distal (MOD) composite by clinician type

**Clinician**	**Appointment time booked MOD composite (mins)**		
	Mean	SD	Range
NHS GD (n=612)	39	10	15-75
Mixed GD (n=191)	43	11	20-105
Private GD (n=505)	46	11	20-100
CDS Dentist (n=115)	43	9	20-60
Therapist (n=75)	44	15	20-120

Appendix Table 7: Composite technique use (N/A= not applicable, i.e. the clinician does not use composite)

**Composite technique**	**% use**				
	**0%**	**1-25%**	**26-75%**	**76-100%**	**N/A**
Rubber dam (n=1501)	32	37	16	12	3
Circumferential metal matrix* (n=1501)	5	14	19	61	1
Sectional metal matrix* (n=1477)	49	16	12	15	7
Circumferential clear matrix* (n=1476)	59	18	9	7	6
Sectional clear matrix* (n=1494)	75	8	3	2	11
Liner (n=1488)	28	45	19	17	1
Wedge* (n=1505)	4	16	21	57	1

*Technique use when restoring a lost proximal surface.

Appendix Table 8: Composite material use (N/A= not applicable, i.e. the clinician does not use composite)

**Composite material**	**% use**				
	**0%**	**1-25%**	**26-75%**	**76-100%**	**N/A**
Bulk-fill flowable only (n=1374)	55	26	5	4	9
Bulk-fill paste only (n=1304)	59	14	8	7	12
Bulk-fill flow & conventional paste (n=1364)	35	24	18	15	8
Bulk-fill flow & bulk-fill paste (n=1264)	68	9	5	3	14
Incremental conventional composite (n=1443)	6	14	20	57	3
Non-incremental conventional composite (n=1254)	63	17	4	2	13

Appendix Table 9: Bonding technique use (N/A= not applicable, i.e. the clinician does not use composite)

**Bonding technique use**	**% use**				
	**0%**	**1-25%**	**26-75%**	**76-100%**	**N/A**
Total-etch 2 step (n=1413)	14	6	7	71	3
Total-etch 3 step (n=1271)	65	6	3	14	12
Selective-etch 1 step (n=1265)	63	11	6	9	11
Selective-etch 2 step (n=1238)	77	5	2	4	14
Selective enamel etch (with selective etch system) (n=1286)	63	10	4	11	12

Appendix Table 10: Clinician reported incidence of sensitivity following direct posterior composite placement by clinician type

**Clinician**	**Sensitivity incidence post composite placement (%)**			
	**0-10%**	**11-25%**	**26-50%**	**51-100%**
NHS GD dentist (%) (n=607)	41	36	17	6
Mixed GD dentist (%) (n=192)	46	34	15	6
Private GD Dentist (%) (n=507)	74	18	5	2
CDS dentist (%) (n=115)	48	33	14	5
Therapist (%) (n=72)	36	32	17	15

Appendix Table 11: Clinician reported incidence of food packing following direct posterior composite placement by clinician type

**Clinician**	**Food packing incidence post composite placement (%)**			
	**0-10%**	**11-25%**	**26-50%**	**51-100%**
NHS GD dentist (%) (n=613)	49	33	12	6
Mixed GD dentist (%) (n=193)	52	32	11	5
Private GD Dentist (%) (n=507)	70	24	5	2
CDS dentist (%) (n=113)	60	30	8	2
Therapist (%) (n=70)	60	21	13	6

Appendix Table 12: Reported incidence of post-operative sensitivity following direct posterior composite placement by years qualified

**Years qualified**	**Sensitivity incidence post composite placement (%)**			
	**0-10%**	**11-25%**	**26-50%**	**51-100%**
0-5 (n=138)	43	30	20	7
6-15 (n=313)	40	36	17	7
16-25 (n=362)	54	29	12	5
≥26 (n=679)	61	26	9	4

Appendix Table 13: Reported incidence of post-operative food packing following direct posterior composite placement by years qualified

**Years qualified**	**Food packing incidence post composite placement (%)**			
	**0-10%**	**11-25%**	**26-50%**	**51-100%**
0-5 (n=138)	53	30	12	5
6-15 (n=311)	53	32	11	4
16-25 (n=367)	58	28	10	4
≥26 (n=680)	61	28	7	4

Appendix Table 14: Reported incidence of post-operative sensitivity following amalgam placement by years qualified

**Years qualified**	**Sensitivity incidence post amalgam placement (%)**			
	**0-10%**	**11-25%**	**26-50%**	**51-100%**
0-5 (n=134)	64	28	5	2
6-15 (n=301)	71	23	4	2
16-25 (n=331)	81	15	3	0
≥26 (n=601)	89	9	1	1

Appendix Table 15: Reported incidence of post-operative food packing following amalgam placement by years qualified

**Years qualified**	**Food packing incidence post amalgam placement (%)**			
	**0-10%**	**11-25%**	**26-50%**	**51-100%**
0-5 (n=134)	69	19	9	2
6-15 (n=300)	75	20	4	1
16-25 (n=333)	85	12	2	1
≥26 (n=601)	92	6	1	1

Appendix Table 16: Experience of use of categories of bulk-fill composites

**Category of bulk-fill composite**	**Experience of use (%)**
Flowable light-cured (n=278)	53
Paste light-cured (n=170)	32
Dual cured (n=32)	6
Non-bulk-fill composite/non-composite (n=40)	8

Appendix Table 17: Opinions on bulk-fill composites in relation to standard composites

**Bulk-fill composites in relation to standard composites**	**Agree/Strongly agree (%)**	**Neither agree nor disagree (%)**	**Disagree/Strongly disagree (%)**
Easier to place (n-1033)	68	26	6
Time-saving (n=1029)	81	16	3
Reduced post-op sensitivity (n=1025)	28	63	9
More predictable (n=1024)	27	60	14
More aesthetic (n=1027)	7	38	55

Appendix Table 18 details the multiple linear regression to explore the influence of various factors on appointment time booked. The significant independent variables and their referents are more thoroughly explained in the text.

Appendix Table 18: Factors related to appointment time booked for direct posterior mesio-occluso-distal (MOD) composite restoration. n=769; $p<0.001$; Adjusted $R^2=0.15$

**Independent variable (predictor)**	**Coefficient**	**Standard error**	**t**	**P>t**	**95% Confidence interval**
**No undergraduate clinical teaching** *(ref had UG teaching)*	0.23	0.93	0.24	0.808	-1.59 - 2.05
**No postgraduate training** *(ref had PG training)*	-0.31	1.25	-0.25	0.802	-2.77 – 2.14
**UK primary dental qualification** *(ref non-UK)*	-1.02	1.22	-0.83	0.404	-3.42 – 1.38
**Type of practice** *(ref NHS general dentist 75-100% NHS patient base)*					
Private general dentist (0-24% NHS patient base)	5.77	1.15	5.04	**0.000**	3.52 – 8.02
Mixed general dentist (25-74% NHS patient base)	3.50	1.24	2.83	**0.005**	1.07 – 5.92
CDS dentist	2.06	1.59	1.29	0.198	-1.07 – 5.18
Therapist	4.83	2.06	2.35	**0.019**	0.79 – 8.88
**Years qualified**	-0.07	0.04	-1.73	0.085	-0.16 – 0.01
**Female** *(ref male)*	-0.32	0.81	-0.39	0.694	-1.91 – 1.27
**Composite user (combined premolar and molar composite usage > 100%)** *(ref combined use <100%)*	-0.48	0.95	-0.51	0.613	-2.35 – 1.39
**Incremental composite user (76-100% use)** *(ref <76% incremental)*	1.92	0.79	2.44	**0.015**	0.37 – 3.45
**Bonding system use** *(ref self-etch 1 step (76-100% use))*					
Total-etch 3 step bond (76-100% use)	3.01	1.46	2.06	**0.040**	0.14 – 5.88
Total-etch 2 step bond (76-100% use)	2.19	1.03	2.12	**0.034**	0.16 – 4.21
Self-etch 2 step bond (76-100% use)	-3.24	2.85	-1.14	0.255	-8.83 – 2.34
**Matrix use** *(ref not CM or SM user)*					
Circumferential metal user (100% use)	0.46	0.87	0.53	0.597	-1.25 – 2.17
Sectional metal user (51-100% use)	3.54	1.13	3.12	**0.002**	1.32 – 5.77
**High wedge use (76-100% use)** *(ref <76% use)*	1.55	0.84	1.84	0.066	-0.10 – 3.21
**Never liner use** *(ref >0% use)*	0.89	0.85	1.05	0.293	-0.77 – 2.55
**Rubber dam use** *(ref 1-75% use)*					
Never	-2.38	0.90	-2.65	**0.008**	-4.14 – -0.62
High (76-100% use)	5.79	1.26	4.61	**0.000**	3.33 – 8.26
**High confidence MOD composite placer** *(ref not high confidence)*	-2.01	0.89	-2.25	**0.024**	-3.76 – -0.26
**Constant**	39.02	1.99	19.65	0.000	35.12 – 42.92

Appendix Table 19 details the multiple linear regression to explore the influence of various factors on private fee charged for an MOD composite.

Appendix Table 19: Factors related to private fee charged for a direct posterior mesio-occluso-distal (MOD) composite restoration. n=711; p<0.0001; adjusted R^2=0.28

Independent variable (predictor)		Coefficient	Standard error	t	P>t	95% confidence interval
**Appointment time booked MOD composite**		1.43	0.16	9.07	**0.000**	1.12 – 1.75
**No undergraduate clinical teaching** *(ref had UG teaching)*		-0.30	4.08	-0.07	0.941	-8.30 – 7.70
**No postgraduate training** *(ref had PG training)*		-2.33	5.63	-0.41	0.679	-13.39 – 8.72
**UK primary dental qualification** *(ref non-UK)*		-7.88	5.28	-1.49	0.136	-18.24 – 2.48
**Type of practice** *(ref NHS general dentist 75-100% NHS patient base)*						
	Private general dentist (0-24% NHS patient base)	27.56	5.11	5.39	**0.000**	17.51 – 37.60
	Mixed general dentist (25-74% NHS patient base)	12.91	5.31	2.43	**0.015**	2.49 – 23.33
	CDS dentist	19.58	10.77	1.82	0.070	-1.57 – 40.73
	Therapist	11.86	9.95	1.19	0.234	-7.69 – 31.40
**Years qualified**		-0.01	0.19	-0.06	0.950	-0.38 – 0.36
**Female** *(ref male)*		-3.64	3.50	-1.04	0.299	-10.52 – 3.24
**Composite user (combined premolar and molar composite usage > 100%)** *(ref combined use <100%)*		1.25	4.39	0.28	0.777	-7.38 – 9.87
**Incremental composite user (76-100% use)** *(ref <76% incremental)*		8.04	3.47	2.32	**0.021**	1.23 – 14.86
**Bonding system use** *(ref self-etch 1 step (76-100% use))*						
	Total-etch 3 step bond (76-100% use)	8.81	6.40	1.38	0.169	-3.76 – 21.38
	Total-etch 2 step bond (76-100% use)	-4.33	4.53	-0.96	0.340	-13.21 – 4.56
	Self-etch 2 step bond (76-100% use)	-3.03	12.43	-0.24	0.808	-27.44 – 21.39
**Matrix use** *(ref not CM or SM user)*						
	Circumferential metal user (100% use)	.571	3.87	0.15	0.883	-7.03 – 8.17
	Sectional metal user (51-100% use)	-7.34	4.89	-1.50	0.134	-16.94 – 2.26
**High wedge use (76-100% use)** *(ref <76% use)*		9.19	3.73	2.46	**0.014**	1.85 – 16.52
**Never liner use** *(ref >0% use)*		1.82	3.65	0.50	0.618	-5.34 – 8.98
**Rubber dam use** *(ref 1-75% use)*						
	Never	-10.53	3.98	-2.65	**0.008**	-18.35 – 2.72
	High (76-100% use)	7.98	5.49	1.45	0.146	-2.79 – 18.76
**High confidence MOD composite placer** *(ref not high confidence)*		8.47	4.01	2.11	**0.035**	0.60 – 16.34
**Constant**		62.36	10.63	5.86	0.000	41.49- 83.24

Appendix Table 20 details the regression to explore the influence of various factors on reported incidence of post-operative sensitivity.

Appendix Table 20: Factors related to low reported incidence of post-operative sensitivity following direct posterior composite placement. n=770; p<0.0001; pseudo R^2=0.11

Independent variable (predictor)	Odds ratio	Standard error	z	P>z	95% confidence interval
**Appointment time booked MOD composite**	1.01	0.01	0.75	0.456	0.99 – 1.02
**No undergraduate clinical teaching** *(ref had UG teaching)*	0.99	0.19	-0.05	0.962	0.68 – 1.45
**No postgraduate training** *(ref had PG training)*	1.21	0.31	0.74	0.457	0.73 – 2.00
**UK primary dental qualification** *(ref non-UK)*	1.00	0.25	0.00	0.997	0.61 – 1.63
**Type of practice** *(ref NHS general dentist 75-100% NHS patient base)*					
Private general dentist (0-24% NHS patient base)	1.50	0.36	1.72	0.085	0.95 – 2.40
Mixed general dentist (25-74% NHS patient base)	0.66	0.17	-1.63	0.103	0.40 – 1.09
CDS dentist	1.14	0.36	0.43	0.670	0.62 – 2.12
Therapist	0.39	0.18	-2.05	**0.040**	0.16 – 0.96
**Years qualified**	1.01	0.01	1.32	0.186	0.99 – 1.03
**Female** *(ref male)*	1.15	0.19	0.81	0.416	0.82 – 1.60
**Composite user (combined premolar and molar composite usage > 100%)** *(ref combined use <100%)*	2.33	0.44	4.48	**0.000**	1.61 – 3.38
**Incremental composite user (76-100% use)** *(ref <76% incremental)*	1.15	0.19	0.82	0.410	0.83 – 1.58
**Bonding system use** *(ref self-etch 1 step (76-100% use))*					
Total-etch 3 step bond (76-100% use)	0.88	0.27	-0.42	0.677	0.48 – 1.62
Total-etch 2 step bond (76-100% use)	0.68	0.15	-1.77	0.076	0.44 – 1.04
Self-etch 2 step bond (76-100% use)	3.15	2.57	1.40	0.160	0.64 – 15.62
**Matrix use** *(ref not CM or SM user)*					
Circumferential metal user (100% use)	1.12	0.20	0.66	0.512	0.79 – 1.59
Sectional metal user (51-100% use)	1.56	0.38	1.81	0.070	0.96 – 2.52
**High wedge use (76-100% use)** *(ref <76% use)*	1.18	0.21	0.96	0.335	0.84 – 1.67
**Never liner use** *(ref >0% use)*	1.75	0.31	3.14	**0.002**	1.23 – 2.49
**Rubber dam use** *(ref 1-75% use)*					
Never	0.88	0.17	-0.66	0.511	0.61 – 1.28
High (76-100% use)	1.05	0.28	0.17	0.868	0.62 – 1.78
**Constant**	0.37	0.18	-2.02	0.043	0.14 – 0.97

Appendix Table 21 details the regression to explore the influence of various factors on incidence of post-operative food packing.

Appendix Table 21: Factors related to low reported incidence of reported post-operative food packing following direct posterior composite placement. A logistic regression analysis; n=768; p<0.0001; pseudo R^2= 0.09

Independent variable (predictor)	Odds ratio	Standard error	z	P>z	95% confidence interval
**Appointment time booked MOD composite**	0.99	0.01	-1.25	0.212	0.98 – 1.01
**No undergraduate clinical teaching** *(ref had UG teaching)*	0.81	0.16	-1.10	0.273	0.55 – 1.18
**No postgraduate training** *(ref had PG training)*	0.94	0.24	-0.25	0.805	0.58 – 1.54
**UK primary dental qualification** *(ref non-UK)*	1.04	0.26	0.15	0.884	0.63 – 1.70
**Type of practice** *(ref NHS general dentist 75-100% NHS patient base)*					
Private general dentist (0-24% NHS patient base)	0.78	0.19	-1.02	0.310	0.48 – 1.26
Mixed general dentist (25-74% NHS patient base)	0.66	0.17	-1.65	0.098	0.40 – 1.08
CDS dentist	0.95	0.31	-0.17	0.867	0.50 – 1.79
Therapist	1.25	0.54	0.51	0.608	0.53 – 2.92
**Years qualified**	1.01	0.01	1.05	0.292	0.99 – 1.03
**Female** *(ref male)*	0.87	0.15	-0.83	0.406	0.63 – 1.21
**Composite user (combined premolar and molar composite usage > 100%)** *(ref combined use <100%)*	2.81	0.56	5.22	**0.000**	1.91 – 4.15
**Incremental composite user (76-100% use)** *(ref <76% incremental)*	1.60	0.27	2.84	**0.005**	1.16 – 2.22
**Bonding system use** *(ref self-etch 1 step (76-100% use))*					
Total-etch 3 step bond (76-100% use)	1.09	0.35	0.27	0.784	0.59 – 2.03
Total-etch 2 step bond (76-100% use)	0.90	0.20	-0.50	0.619	0.58 – 1.38
Self-etch 2 step bond (76-100% use)	2.25	1.56	1.18	0.239	0.58 – 8.72
**Matrix use** *(ref not CM or SM user)*					
Circumferential metal user (100% use)	0.89	0.16	-0.67	0.504	0.63 – 1.25
Sectional metal user (51-100% use)	2.48	0.64	3.51	**0.000**	1.49 – 4.12
**High wedge use (76-100% use)** *(ref <76% use)*	1.17	0.20	0.91	0.361	0.83 – 1.64
**Never liner use** *(ref >0% use)*	1.07	0.19	0.38	0.705	0.76 – 1.51
**Rubber dam use** *(ref 1-75% use)*					
Never	0.81	0.15	-1.17	0.240	0.56 – 1.16
High (76-100% use)	1.42	0.40	1.23	0.219	0.81 – 2.48
**Constant**	0.98	0.48	-0.04	0.972	0.37 – 2.58
